# Differential etiopathogenic features of vulvar squamous cell carcinomas in sub‐Saharan Africa and Europe

**DOI:** 10.1002/ijc.34314

**Published:** 2022-10-31

**Authors:** Natalia Rakislova, Nuria Carreras‐Dieguez, Carolina Manzotti, Ofelia Saúde, Marta del Pino, Laurina Chulo, Ricardina Rangeiro, Lucilia Lovane, Cesaltina Lorenzoni, Fabiola Fernandes, Maria Teresa Rodrigo‐Calvo, Sherley Diaz‐Mercedes, Inmaculada Ribera‐Cortada, Esther Sanfeliu, Ricardo López del Campo, Lorena Marimon, Silvia Alós, Naiara Vega, Francisco M. Pérez, Isabel Trias, Carla Carrilho, Jaume Ordi

**Affiliations:** ^1^ Facultat de Medicina i Ciències de la Salut, c. Casanova Universitat de Barcelona (UB) Barcelona Spain; ^2^ Department of Pathology, Hospital Clínic of Barcelona University of Barcelona Barcelona Spain; ^3^ Department of Pathology, Barcelona Institute of Global Health (ISGlobal) Hospital Clínic—Universitat de Barcelona Barcelona Spain; ^4^ Department of Obstetrics and Gynecology Hospital Clínic—Universitat de Barcelona Barcelona Spain; ^5^ Department of Pathology Hospital Central de Maputo Maputo Mozambique; ^6^ Institut d'Investigacions Biomèdiques August Pi i Sunyer (IDIBAPS) Barcelona Spain; ^7^ Department of Obstetrics and Gynecology Hospital Central de Maputo Maputo Mozambique; ^8^ Department of Pathology Universidade Eduardo Mondlane Maputo Mozambique

**Keywords:** HPV, Mozambique, p16, p53, Spain, vulvar cancer

## Abstract

Two pathways have been described for vulvar squamous cell carcinomas (VSCC), one associated with human papillomavirus (HPV), and the other HPV‐independent. We compared the etiopathogenic features of a series of VSCC from Mozambique, a sub‐Saharan country with high prevalence of HPV and HIV, with those of Spain, a European country with low prevalence of HPV and HIV. All VSCC diagnosed at the two institutions from January 2018 to December 2020 were included (n = 35 and n = 41, respectively). HPV DNA detection and genotyping, and immunohistochemistry for p16 and p53 were performed. Tumors showing p16 positive staining and/or HPV DNA positivity were considered HPV‐associated. 34/35 tumors (97%) from Mozambique and 8/41 (19%) from Spain were HPV‐associated (*P* < .001). Mean age of the patients from Mozambique and Spain was 45 ± 12 and 72 ± 14, respectively (*P* < .001). No differences were found in terms of HPV genotypes or multiple HPV infection rates. 1/35 tumors (3%) from Mozambique and 29/41 (70%) from Spain showed abnormal p53 immunostaining (*P* < .001). In contrast with the predominance of HPV‐independent VSCC affecting old women in Europe, most VSCC in sub‐Saharan Africa are HPV‐associated and arise in young women. This data may have important consequences for primary prevention of VSCC worldwide.

## Graphical abstract

Vulvar squamous cell carcinoma (VSCC) can arise from HPV or non‐HPV causes, and that affects prognosis. Here, the authors compared VSCC samples from a population in Mozambique, where HPV prevalence is high, with those from Spain, where it is low. In Mozambique, 97% of VSCC were HPV‐associated, based on p16 staining and HPV DNA positivity, compared with only 19% of VSCC in Spain. Mean age of patients in Mozambique was 45, vs 72 in Spain. Vaccination programs, the authors suggest, could therefore help prevent the majority of VSCC in sub‐Saharan Africa.

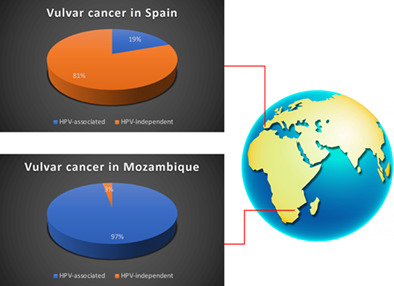

## 1 INTRODUCTION

Human papillomavirus (HPV) is a well‐established cause of a variety of human cancers, which include anogenital neoplasms (cervix, anal canal, vulva, vagina and penis) as well as head and neck tumors.^1^ In contrast with the uterine cervix, where HPV is etiologically involved in the vast majority of cancers,^1^ in other anatomical sites, such as the head and neck, the penis or the vulva, a second, HPV‐independent, etiopathogenic pathway is responsible of a significant proportion of tumors. In all these sites, both HPV‐associated and HPV‐independent tumors are squamous cell carcinomas (SCCs), and this histological type represents over 90% of the malignant neoplasms. In vulvar squamous cell carcinomas (VSCC), it is traditionally accepted that basaloid or warty subtypes are associated with HPV,^2^ while keratinizing variants tend to arise independently of HPV infection.^3^ However, pure histological criteria have shown limitations in classifying a VSCC as HPV‐associated or independent.^4^ Interestingly, a number of studies have consistently shown that, in practically all the anatomical sites, HPV‐associated tumors have better prognosis than HPV‐independent carcinomas.^5^, ^6^ Due to this prognostic relevance and the consequences in terms of possible prevention related to HPV vaccination programs, the last revisions of the WHO classifications of cancers of the vulva,^7^ vagina,^7^ the penis,^8^ or the head and neck^9^ separate SCCs into two main categories, HPV‐associated and HPV‐independent. Due to the limitations of pure histological criteria and the assumed sensitivity and specificity of molecular techniques of HPV detection^10^ and p16 immunohistochemical (IHC) staining, a surrogate biomarker of HPV status,^11^ HPV detection and/or p16 IHC are considered by the World Health Organization (WHO) as essential diagnostic criteria for VSCC.

The proportion of HPV‐associated and ‐independent VSCC is variable depending on the geographic area.^10^ Several studies, mainly conducted in Europe and USA have shown that most vulvar tumors arise independently of HPV. These studies show percentages of HPV‐associated VSCC that range from 15% to 20% in Europe to 40% to 50% in the USA.^4^, ^10^ However, in contrast with the relatively well‐known epidemiology of vulvar cancer in these high‐income sites, there is very scant information on the etiopathogenic features of VSCC in many low‐income areas and particularly, in sub‐Saharan Africa. Indeed, the pivotal study published by de Sanjose et al comprising over 1700 carcinomas of the vulva included only a very minor subset of tumors from sub‐Saharan Africa.^10^ Remarkably, these regions have marked differences in terms of HPV prevalence, which may result in pronounced variations in the proportion of HPV‐associated and ‐independent tumors. Moreover, some of these regions have a high proportion of people living with HIV frequently associated with severe acquired immunodeficiency syndrome (AIDS),^12^ a condition that increases the risk of persistent HPV‐infections and HPV‐associated carcinomas.^13^

In this study, we compared the etiopathogenic features of a series of VSCC from Mozambique, a sub‐Saharan country in South‐East Africa with high prevalence of HPV and HIV/AIDS, with those of Spain, a country in South‐Western Europe with relatively low prevalence of HPV and HIV.

## 2 METHODS

### 2.1 Case selection

The files of the departments of Pathology and the hospital‐based cancer registry of the Maputo Central Hospital, a 1500‐bed hospital that is the only quaternary care center in Mozambique and a national referral center in Maputo, Mozambique, and the Hospital Clínic of Barcelona, a 700‐bed tertiary referral center in Barcelona, Spain, from January 2018 to December 2020 were reviewed. All cases diagnosed as VSCC during this period were retrieved, and the available material was reviewed. All cases fulfilling the following inclusion criteria were included in the study: (1) presence of invasive VSCC in the vulva and (2) available material for histological revision and HPV detection by polymerase chain reaction (PCR) and p16 IHC in the invasive tumor.

### 2.2 Histological revision and review of the clinical charts

Hematoxylin and eosin sections of all tumors were reviewed. In the histological revision, the presence of invasive carcinoma was confirmed, the histological variant of the tumor was assessed (keratinizing, nonkeratinizing, basaloid, warty, verrucous, etc.) and the most representative and well‐preserved paraffin‐embedded block was selected for HPV detection and IHC staining.

All the histological slides were evaluated by two gynecological pathologists with expertise in vulvar pathology (NR, JO). The observers were aware of the geographical origin of the cases but were blind to the HPV testing results.

The clinical charts form all patients were reviewed.

### 2.3 Tissue preparation, nucleic‐acid isolation and HPV‐DNA detection

DNA extraction was performed on whole sections of formalin‐fixed paraffin‐embedded tissue from surgical specimens or vulvar biopsies as previously described. In all cases, the analyzed tissue included the invasive tumor, but no microdissection was performed. Sectioning and sample preparation were carried out with the highest safety measures to avoid cross‐contamination.

HPV DNA detection and typing were performed using SPF10 PCR and the LiPA25 system (version 1, Labo Biomedical Products, Rijswijk, The Netherlands). A volume of 10 μl of isolated DNA was used for PCR amplification through the SPF10‐LiPA system (Fujirebio, Gent, Belgium). HPV genotyping was conducted using INNO‐LiPA HPV Genotyping Extra II kit (Fujirebio, Gent, Belgium). This system allows the amplification and genotyping of high‐risk HPV 16, 18, 31, 33, 35, 39, 45, 51, 52, 56, 58, 59 and 68; of the probable high‐risk HPV 26, 53, 66, 70, 73 and 82; and of low‐risk HPVs 6, 11, 40, 42, 43, 44, 54, 61, 62, 67, 81, 83 and 89. Quality in each run was confirmed with both positive and negative controls for DNA isolation, amplification, hybridization and genotyping.

### 2.4 p16 and p53 IHC

All cases were stained with a p16 monoclonal antibody using the CINtec Histology Kit (clone E6H4; Roche‐mtm‐Laboratories, Heidelberg, Germany). Tumors showing strong and diffuse block‐like staining were considered as positive (p16 upregulation), whereas patchy or completely negative p16 staining was considered as p16 negative.^11^, ^14^

All cases were stained for p53 with the monoclonal antibody (clone DO‐7; Dako, Carpinteria, CA). The IHC staining was evaluated in the invasive tumor following the recent p53 pattern‐based interpretation framework,^15^, ^16^ which includes six major categories: two normal (wild‐type) and four abnormal (mutant) patterns.

Normal patterns, suggestive of wild‐type p53 protein included: (1) scattered (occasional positive nuclei in the basal and/or parabasal layer) and (2) mid‐epithelial staining with basal sparing (moderate to strong nuclear p53 staining in the parabasal layers staining, with sparing of the basal cells).

Abnormal p53 staining patterns, suggestive of mutant protein included: (1) basal overexpression (continuous, strong staining of the nuclei in the basal layer), (2) diffuse (parabasal) overexpression (continuous, strong staining of the nuclei in the basal layer with suprabasal extension of the positive cells), (3) cytoplasmic (cytoplasmic staining with or without nuclear expression) and (4) null patterns (complete absence of staining in the tumor, with evidence of intrinsic positive control in the adjacent skin, stromal or inflammatory cells).^15^, ^16^

All IHC stains were performed using the automated BenchMark ULTRA platform (Ventana, Tucson, AZ). All IHC and molecular analysis were performed at the pathology department of the Hospital Clinic in Barcelona.

### 2.5 Criteria for classifying a tumor as HPV‐associated or HPV‐independent

All tumors showing positive p16 IHC staining, independently of the results of the HPV detection and typing, or positive result of the HPV detection, independently of the results of p16 IHC, were considered as HPV‐associated, whereas cases negative for both techniques were considered as HPV‐independent. Histological features and p53 IHC staining were not considered for classifying a tumor as HPV‐associated or independent.

### 2.6 Statistical analysis

Data analyses were performed with the SPSS 23.0 statistical package (SPSS, Chicago, IL). Categorical variables were expressed as absolute numbers and percentages. The chi‐square exact test was used to compare qualitative variables. Quantitative variables were expressed as mean ± SD, and the Student's *t* or analysis of variance tests were used for comparisons.

## 3 RESULTS

### 3.1 General features

From January 2018 to December 2020, 41 VSCC were diagnosed at the Maputo Central Hospital (Mozambique) and 41 at the Hospital Clínic of Barcelona (Spain). From the 41 cases initially diagnosed in Mozambique, four were excluded because of the absence of invasive carcinoma, and two because of the absence of sufficient material for further analysis. None of the cases from Spain were excluded. Thus, finally 35 patients from Mozambique and 41 from Spain fulfilled the inclusion criteria. Mean age of the patients from Mozambique and Spain was 45 ± 12 and 72 ± 14, respectively (*P* < .001). Tables 1 and 2 show the age and HIV status of the patients form Mozambique and Spain, respectively. The information of the HIV status was available in only nine out of the 35 patients form Mozambique, but seven of them (78%) were positive. All patients from Spain were HIV negative. Histologically, 12 tumors from Mozambique were basaloid, six were nonkeratinizing, four were warty and 13 keratinizing. In the Spanish series, seven were basaloid, one were nonkeratinizing, three were warty and 30 keratinizing (including a verrucous carcinoma). The distribution of histological variants was significantly different between the countries (*P* = .008).

**TABLE 1:** Age, histological variant, HPV‐DNA result, p16 and p53 IHC of the tumors from Mozambique

Case	Age	HIV	Histological variant	p16	HPV‐DNA	p53
M1	43	−	Keratinizing	+	Negative	Wild type
M2	28	NA	Basaloid	+	16	Wild type
M3	50	NA	Nonkeratinizing	+	X	Wild type
M4	43	NA	Nonkeratinizing	+	31‐33‐52‐58‐70	Wild type
M5	51	+	Basaloid	+	16	Wild type
M6	39	NA	Keratinizing	+	Negative	Wild type
M7	32	NA	Nonkeratinizing	+	Negative	Wild type
M8	38	NA	Warty	−	6	Wild type
M9	60	NA	Basaloid	+	16‐58	Wild type
M10	58	−	Keratinizing	+	16	Wild type
M11	75	NA	Warty	+	18	Wild type
M12	40	NA	Basaloid	+	18‐52‐56‐58‐82	Wild type
M13	37	NA	Keratinizing	+	16	Wild type
M14	33	NA	Keratinizing	+	33	Wild type
M15	49	+	Keratinizing	+	16‐51	Mutated
M16	66	NA	Keratinizing	+	18	Wild type
M17	44	+	Keratinizing	+	Negative	Wild type
M18	41	NA	Basaloid	+	Negative	Wild type
M19	26	NA	Keratinizing	+	16	Wild type
M20	39	+	Basaloid	+	Negative	Wild type
M21	33	NA	Keratinizing	+	16	Wild type
M22	45	NA	Warty	+	33‐58‐68‐82	Wild type
M23	50	NA	Basaloid	+	33	Wild type
M24	45	NA	Nonkeratinizing	+	Negative	Wild type
M25	42	NA	Basaloid	+	33	Wild type
M26	32	NA	Nonkeratinizing	+	Negative	Wild type
M27	80	NA	Keratinizing	−	Negative	Wild type
M28	52	NA	Keratinizing	+	Negative	Wild type
M29	38	NA	Basaloid	+	Negative	Wild type
M30	54	NA	Basaloid	+	33	Wild type
M31	41	+	Keratinizing	+	X	Wild type
M32	28	NA	Basaloid	+	31‐58	Wild type
M33	44	+	Warty	+	16‐52	Wild type
M34	45	+	Nonkeratinizing	+	Negative	Wild type
M35	49	NA	Basaloid	+	Negative	Wild type

Abbreviation: NA, not available.

**TABLE 2:** Age, histological variant, HPV‐DNA result, p16 and p53 IHC of the tumors from Spain

Case	Age	HIV	Histological variant	p16	HPV‐DNA	p53
S1	69	−	Basaloid	+	Negative	Wild type
S2	83	−	Keratinizing	−	Negative	Wild type
S3	92	−	Keratinizing	−	Negative	Mutated
S4	84	−	Keratinizing	−	Negative	Wild type
S5	47	−	Keratinizing	−	Negative	Mutated
S6	86	−	Keratinizing	−	Negative	Mutated
S7	86	−	Verrucous	−	Negative	Wild type
S8	58	−	Basaloid	+	33	Wild type
S9	61	−	Keratinizing	−	Negative	Wild type
S10	73	−	Keratinizing	+	56	Wild type
S11	53	−	Keratinizing	−	Negative	Mutated
S12	77	−	Keratinizing	−	Negative	Mutated
S13	50	−	Basaloid	+	16	Wild type
S14	58	−	Warty	+	16‐53	Wild type
S15	81	−	Keratinizing	−	Negative	Mutated
S16	61	−	Keratinizing	−	Negative	Mutated
S17	78	−	Keratinizing	−	Negative	Mutated
S18	82	−	Keratinizing	−	Negative	Mutated
S19	82	−	Warty	−	Negative	Mutated
S20	69	−	Keratinizing	−	Negative	Mutated
S21	82	−	Keratinizing	−	Negative	Wild type
S22	78	−	Keratinizing	−	Negative	Mutated
S23	54	−	Basaloid	+	16	Wild type
S24	78	−	Warty	−	Negative	Mutated
S25	74	−	Basaloid	+	6,16	Mutated
S26	87	−	Keratinizing	−	Negative	Mutated
S27	90	−	Keratinizing	−	Negative	Mutated
S28	62	−	Keratinizing	−	Negative	Mutated
S29	85	−	Keratinizing	−	Negative	Mutated
S30	90	−	Keratinizing	−	Negative	Mutated
S31	58	−	Keratinizing	−	Negative	Mutated
S32	64	−	Keratinizing	−	Negative	Mutated
S33	70	−	Nonkeratinizing	−	Negative	Mutated
S34	90	−	Keratinizing	−	Negative	Mutated
S35	67	−	Keratinizing	−	Negative	Mutated
S36	82	−	Basaloid	−	Negative	Mutated
S37	90	−	Keratinizing	−	Negative	Mutated
S38	46	−	Keratinizing	−	Negative	Mutated
S39	50	−	Keratinizing	+	33	Wild type
S40	88	−	Keratinizing	−	Negative	Mutated
S41	47	−	Keratinizing	−	Negative	Mutated

### 3.2 Association with HPV‐DNA and p16 IHC results

Thirty‐four out of 35 tumors (97.1%) from Mozambique and 8/41 (19.5%) from Spain were HPV‐associated (*P* < .001).

In the series from Mozambique HPV‐DNA was positive in 22 (62.9%) of the tumors. p16 IHC was positive in 33/35 tumors (94.3%), 12 (36.4%) of whom had negative HPV‐DNA. Only one tumor positive for HPV‐DNA showed negative p16 IHC. In this tumor HPV6 was identified. HPV16 (43%), HPV33 (29%), HPV58 (24%) and HPV18 (14%) were the HPV types more frequently identified in Mozambique. Seven tumors showed infection by multiple HPV types. Table 1 shows the histological variant, HPV‐DNA result, as well as p16 and p53 IHC staining of the tumors from Mozambique.

**FIGURE 1:**
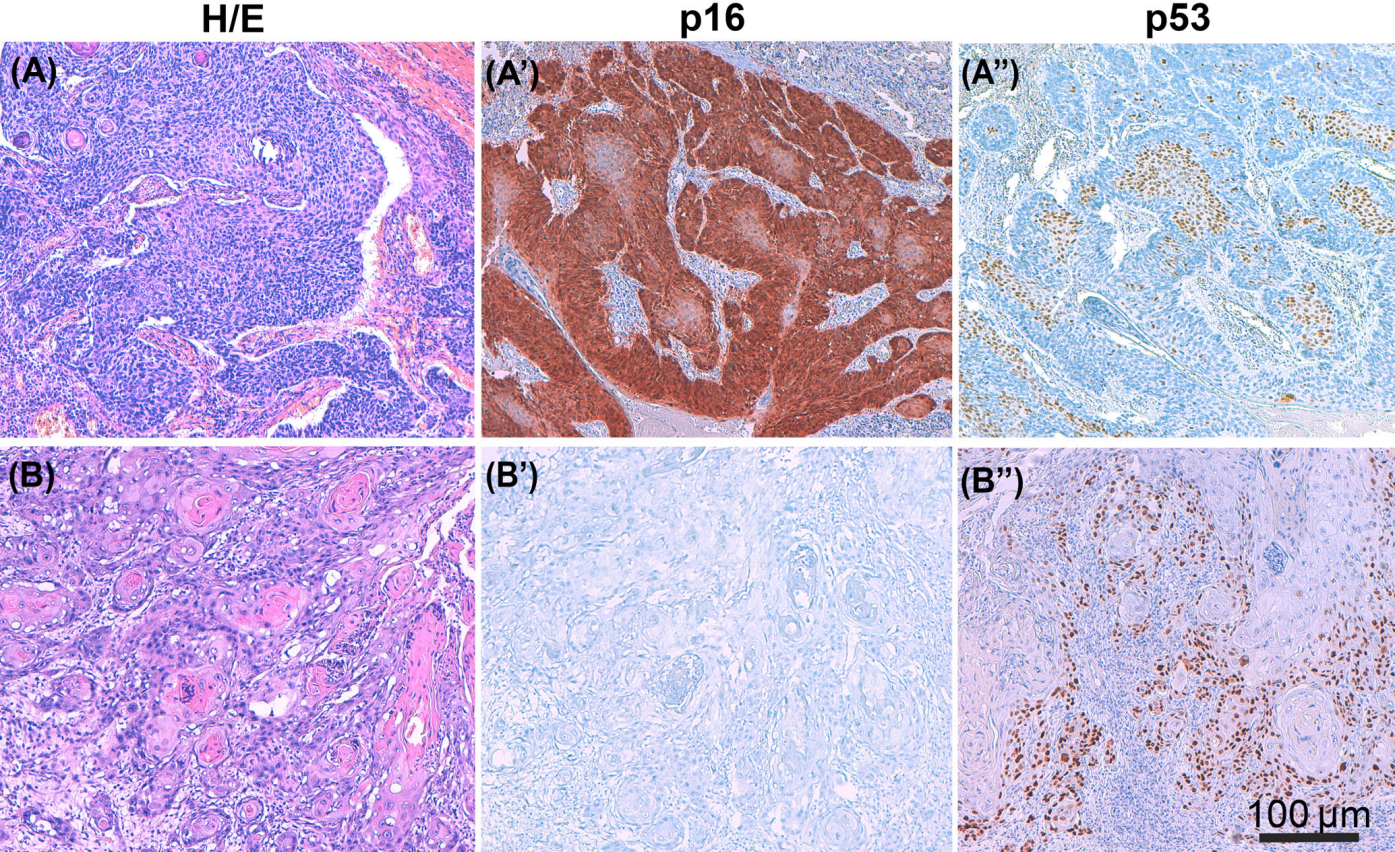
A characteristic example of each of the two types of vulvar squamous cell carcinoma (VSCC), HPV‐associated and HPV‐independent. The former being predominant in sub‐Saharan countries such as Mozambique and the latter in European countries such as Spain. (A) HPV‐associated invasive VSCC (H&E ×200) with basaloid histology, positive p16 staining (A') and wild‐type p53 staining showing mid‐epithelial pattern (A"). (B) HPV‐independent invasive VSCC (H&E ×200) with keratinizing histology, negative p16 staining (B') and abnormal p53 staining showing overexpression pattern (B")

In the series from Spain HPV‐DNA was positive in 7 (17.1%) of the tumors. p16 IHC was positive in 8/41 tumors (19.5%), and only one of them (12.5%) had negative HPV‐DNA. No tumors were negative for p16 and positive for HPV‐DNA. HPV16 (50%), HPV33 (25%), HPV53 (7%) and HPV56 (7%) were the HPV types more frequently identified in Spain. Two tumors showed infection by multiple HPV types. Table 2 shows the histological variant, HPV‐DNA result, p16 and p53 IHC of the tumors from Spain. Figure 1 illustrates histological and IHC features of each of the two types of VSCC, HPV‐associated and HPV‐independent.

No significant differences were found in terms of percentage of HPV genotypes between the two countries.

### 3.3 p53 IHC results

One out of 35 tumors (3%) from Mozambique and 29/41 (70%) from Spain showed abnormal pattern of p53 immunostaining (*P* < .001).

## 4 DISCUSSION

Our study provides relevant data on the epidemiology of VSCC in sub‐Saharan Africa, a geographical region where this information is almost nonexistent. Our series highlight the exceptionally high prevalence of HPV in VSCC in Mozambique and confirms that marked etiopathogenic differences exist between VSCC in sub‐Saharan Africa and Europe. Thus, over 95% of the VSCC in Mozambique but only 19% of the VSCC from Spain were HPV‐associated. In accordance with these etiopathogenic differences, the clinical presentation was also markedly different, with patients from Spain being almost three decades older in average than patients from Mozambique.

The findings observed in our series from Spain are in keeping with most series reported from high‐income countries,^6^, ^10^, ^14^ where VSCC is typically a disease of older women, and arise independently from HPV, in association with inflammatory and reactive lesions of the vulvar skin, such as lichen sclerosus and lichen simplex chronicus.

Contrarily, the sub‐Saharan African series showed a completely different epidemiological pattern, with almost all tumors arising via an HPV‐associated pathway. A high prevalence of ano‐genital HPV infections has been reported in sub‐Saharan African women.^17^, ^18^ Indeed, cancer of the uterine cervix, the most frequent malignant tumor associated with HPV, is the most common cancer in women in sub‐Saharan Africa, with the highest incidence in Eastern and Southern Africa^19^, ^20^, ^21^ and an extremely high prevalence has been reported by our group in Southern Mozambique.^22^ Our results show that, although the current WHO 2020 classification separates VSCC into HPV‐associated and HPV‐independent tumors and recommends the use of HPV testing and/or p16 IHC for the proper classification, this requirement should probably be considered unnecessary in many sub‐Saharan countries, where the number of HPV‐independent tumors is likely to be extremely low, and the resources and laboratory capacities are, in general, particularly limited.^23^

In addition to the high prevalence of HPV infection, the high prevalence of HIV infection in Mozambique^24^ has been proposed as the most likely explanation for the increase in the incidence in cancer of the uterine cervix observed in Mozambique and several sub‐Saharan countries.^22^ According to the UNAIDS Global Report, the prevalence of HIV in Mozambique was as high as 45% in women aged 28 to 47 in a community‐based study in a rural district of Maputo province,^25^ with a high number of patients not receiving antiretroviral treatment and presenting with AIDS.^26^ People living with HIV are at a high risk of developing HPV‐related cancers,^27^ and both HPV and HIV type 1 are classified as carcinogens by the International Agency for Research on Cancer (IARC).^28^ However, in contrast to the relatively well‐known association between HIV and cervical cancer, studies assessing the risk of developing VSCC among people living with HIV are missing. Remarkably, 78% of the patients from Mozambique in whom the information was available were HIV positive, which was in contrast with the 0% of HIV‐positive patients in the Spanish cohort.

The results of our study, showing a relatively high proportion of tumors strongly positive for p16 but negative for HPV‐DNA, suggest that p16 IHC is more robust and reliable than HPV‐DNA detection in formalin‐fixed, paraffin‐embedded material, which is the type of sample usually available for many solid tumors such as VSCC.^4^ The finding of wild‐type p53 IHC staining, the usual pattern in HPV‐associated VSCC,^15^ in all tumors from Mozambique contrasts with the high proportion of VSCC from Spain with an abnormal p53 IHC staining, the commonest IHC staining in HPV‐independent carcinomas.^15^, ^28^ These results support a strong association with HPV in Mozambican cohort, despite negative results for HPV testing. This data is also in keeping with previous work by our group on a large series of VSCC showing that, although in most cases p16 IHC and HPV PCR testing results were concordant (ie, both negative or both positive), in a small percentage of VSCC discordant p16/HPV testing results were observed. Interestingly, the subset of tumors showing p16 positive IHC staining but negative HPV PCR testing result shared marked similarities with the tumors positive for both p16 and HPV, in terms of mean age (younger patients) and histological distribution of the tumors (high percentage of basaloid/warty tumors), whereas the subset of tumors showing p16 negative IHC staining and positive HPV PCR testing result shared marked similarities with the tumors negative for both p16 and HPV, in terms of mean age (older patients) and histological distribution of the tumors (marked predominance of keratinizing tumors), indicating that p16 is more robust than HPV detection in formalin fixed, paraffin embedded tissues.^4^

Although most of the cases showing p53 IHC abnormal patterns of staining suggestive of mutation were HPV‐independent VSCC, a small subset of HPV‐associated tumors showed these abnormal patterns of p53 staining. This finding has already been reported in studies based on IHC, as well as in studies analyzing genetic mutations.^4,15,16^ Indeed, almost all the mutational differences between HPV‐associated and HPV‐independent VSCC are quantitative and not qualitative, as almost all mutations have been described in the two types of tumors, but with different frequencies.

In conclusion, the majority of VSCC in Mozambique, a country in South‐Eastern Africa probably representative of the situation of many sub‐Saharan African countries, are HPV‐associated and arise in young women, which markedly contrasts with the predominance of HPV‐independent VSCC affecting old women observed in Europe. This data may have important consequences for primary prevention policies of VSCC worldwide, and suggest that, whereas vaccination programs could prevent a small proportion of VSCC in many western countries, they can protect sub‐Saharan African women of the vast majority of VSCC.

## AUTHOR CONTRIBUTIONS

Conceptualization, Jaume Ordi, Natalia Rakislova, Marta del Pino and Carla Carrilho; methodology, Lorena Marimon, Silvia Alós, Naiara Vega, Francisco M. Pérez; investigation Nuria Carreras‐Dieguez, Carolina Manzotti, Ofelia Saúde, Laurina Chulo, Ricardina Rangeiro, Lucilia Lovane, Cesaltina Lorenzoni, Sherley Diaz‐Mercedes, Inmaculada Ribera‐Cortada, Esther Sanfeliu, Ricardo López del Campo, Fabiola Fernandes, Maria Teresa Rodrigo‐Calvo, Isabel Trias, Carla Carrilho, Natalia Rakislova and Jaume Ordi; data curation, Natalia Rakislova, Nuria Carreras‐Dieguez, Jaume Ordi and Carla Carrilho; formal analysis, Marta del Pino; writing‐original draft preparation, Jaume Ordi, Natalia Rakislova, Marta del Pino and Nuria Carreras‐Dieguez; writing‐review and editing, Ofelia Saúde, Carolina Manzotti, Laurina Chulo, Ricardina Rangeiro, Lucilia Lovane, Cesaltina Lorenzoni, Sherley Diaz‐Mercedes, Inmaculada Ribera‐Cortada, Esther Sanfeliu, Ricardo López del Campo, Fabiola Fernandes, Isabel Trias, Lorena Marimon, Silvia Alós, Naiara Vega, Francisco M. Pérez, Maria Teresa Rodrigo‐Calvo, Nuria Carreras‐Dieguez and Carla Carrilho; visualization, Jaume Ordi, Natalia Rakislova, Nuria Carreras‐Dieguez and Marta del Pino. The work reported in the paper has been performed by the authors, unless clearly specified in the text.

## FUNDING INFORMATION

Project PI17/00772 funded by Instituto de Salud Carlos III and co‐funded by the European Union (ERDF) “A way to make Europe.”

## CONFLICT OF INTEREST

The authors declare no conflicts of interest.

## ETHICS STATEMENT

The study was approved by the Ethics Committees of the Faculty of Medicine of Maputo Central Hospital (CIBS FM&HCM/071/2017) and Hospital Clinic of Barcelona (ref HCB/2020/1198).

**Abbreviations**
AIDS: acquired immunodeficiency syndrome
HPV: human papillomavirus
IHC: immunohistochemical
PCR: polymerase chain reaction
SCC: squamous cell carcinomas
VSCC: vulvar squamous cell carcinoma
WHO: World Health Organization

## Data Availability

The data that support the findings of this study are available from the corresponding author upon reasonable request.
